# Implementation of Epigenetic Variation in Sorghum Selection and Implications for Crop Resilience Breeding

**DOI:** 10.3389/fpls.2021.798243

**Published:** 2022-01-27

**Authors:** Dikungwa Ketumile, Xiaodong Yang, Robersy Sanchez, Hardik Kundariya, John Rajewski, Ismail M. Dweikat, Sally A. Mackenzie

**Affiliations:** ^1^Department of Agronomy and Horticulture, University of Nebraska–Lincoln, Lincoln, NE, United States; ^2^Department of Biology, The Pennsylvania State University, University Park, PA, United States; ^3^Department of Biology and Plant Science, The Pennsylvania State University, University Park, PA, United States

**Keywords:** *Sorghum bicolor*, *MutS HOMOLOG 1*, phenotypic plasticity, nitrogen stress, G×E

## Abstract

Crop resilience and yield stability are complex traits essential for food security. *Sorghum bicolor* is an important grain crop that shows promise for its natural resilience to drought and potential for marginal land production. We have developed sorghum lines in the Tx430 genetic background suppressed for *MSH1* expression as a means of inducing *de novo* epigenetic variation, and have used these materials to evaluate changes in plant growth vigor. Plant crossing and selection in two distinct environments revealed features of phenotypic plasticity derived from *MSH1* manipulation. Introduction of an epigenetic variation to an isogenic sorghum population, in the absence of selection, resulted in 10% yield increase under ideal field conditions and 20% increase under extreme low nitrogen conditions. However, incorporation of early-stage selection amplified these outcomes to 36% yield increase under ideal conditions and 64% increase under marginal field conditions. Interestingly, the best outcomes were derived by selecting mid-range performance early-generation lines rather than highest performing. Data also suggested that phenotypic plasticity derived from the epigenetic variation was non-uniform in its response to environmental variability but served to reduce genotype × environment interaction. The MSH1-derived growth vigor appeared to be associated with enhanced seedling root growth and altered expression of auxin response pathways, and plants showed evidence of cold tolerance, features consistent with observations made previously in Arabidopsis. These data imply that the MSH1 system is conserved across plant species, pointing to the value of parallel model plant studies to help devise effective plant selection strategies for epigenetic breeding in multiple crops.

## Introduction

Plant phenotype is conditioned by the combination of genes present and epigenetically controlled expression patterns during development and in response to environmental changes (He and Li, 2018). Crop breeding relies on the available genetic variation within a plant species, and that which can be introduced *trans-* and *cis*genically, to advance a lineage through recombination and recurrent selection toward improved trait combinations (Xu et al., 2017). However, to date, breeding programs have not exploited the potential of epigenetic variation for enhancing plant performance, even though evidence suggests that epigenetic processes are important to the ability of a plant to adapt to environmental fluctuations. One reason for the delay in implementation of epigenetic selection in a crop breeding strategy may be concern that epigenetic variation is inherently unstable and refractory to practical selection procedures (Gallusci et al., 2017; Springer and Schmitz, 2017).

Our group has conducted studies to assess the potential for incorporation of epigenetic variation to crop selection procedures by incorporating a system that induces *de novo* epigenetic variation in plants. *MutS HOMOLOG 1* (*MSH1*) is a plant-specific nuclear gene that influences organellar genomic stability functions in plants (Abdelnoor et al., 2003; Davila et al., 2011; Xu et al., 2011). Disruption of *MSH1* in the plastid results in plastid genome instability that is accompanied by nuclear epigenetic reprogramming (Virdi et al., 2015, 2016; Beltrán et al., 2018). Outcomes of these *de novo* epigenetic effects include induction of transgenerational stress memory (Xu et al., 2012; Yang et al., 2020) and enhancement of resilience and growth vigor following crossing (Virdi et al., 2015) and grafting (Kundariya et al., 2020) of the *msh1* mutant. Various derived epigenetic states, which occur in all plants tested to date (Xu et al., 2012), serve as valuable materials for understanding plant epigenetic phenomena (Kundariya et al., 2020; Yang et al., 2020) and offer strategies for exploiting non-genetic variation in a practical plant breeding context (Yang et al., 2015; Raju et al., 2018a).

*Sorghum bicolor* is a grass species important for its production of grain for human consumption, animal feed, and biofuel production. Agronomic sorghum types range from production of grain to sucrose to biomass (Mullet et al., 2014), and the crop can be bred for perennial cultivation (Cox et al., 2018). Sorghum can be adapted to areas with low rainfall (Borrell et al., 2014) and has a potential for production on marginal lands (Boyles et al., 2017). This demonstrated versatility suggests that sorghum has a capacity for phenotypic plasticity, making it an excellent candidate for studies on epigenetic potential.

Previously, we have introduced an MSH1-RNAi transgene construct to the sorghum line Tx430 to investigate the phenotypic effects of MSH1 suppression in this species (Santamaria et al., 2014). The studies resulted in a strikingly slow-growing, abundant-tillering *msh1* phenotype in sorghum, evidence of heritable, non-genetic *msh1* memory effects in derived transgene-null progeny, and enhanced growth potential following crosses of wild-type isogenic selections to *msh1* memory lines. We also observed a remarkable variation in plant height in the derived Tx430 × Tx430*_*msh1*_* F_2_ and F_3_ progeny populations, implying enhanced reversion of a dwarfing gene in this line and complicating assessment of epigenetic variation versus potential genetic instability (Santamaria et al., 2014).

Here, we present a more detailed investigation of Tx430*_*msh1*_* effects and their potential utility in crop breeding. Using a *de novo*-synthesized Epi population, we show that epigenetic reprogramming by *MSH1* suppression has no effect on the stability of *dw3*, a dwarfing mutation present in Tx430 (Miller, 1984; Farfan Barrero et al., 2012). Incorporation of the *msh1* effect to Tx430 and systematic tracking of individual lines derived from crossing wild-type and *msh1*-modified Tx430 produced evidence of a significant non-genetic and heritable phenotype variation. Comparison of selection and non-selection strategies within these populations impacted yield outcomes significantly under both ideal and marginal (low nitrogen) field conditions, indicating that the epigenetic variation induced by *msh1* has value in plant performance and resilience. We also show evidence of similarities in *msh1* effects across plant species and posit that parallel investigation of the *MSH1* system in model plant species can directly inform the design of breeding strategies appropriate across multiple crop species.

## Results

### Assessing MSH1 Effect on Genetic Reversion Rate

**FIGURE 1:**
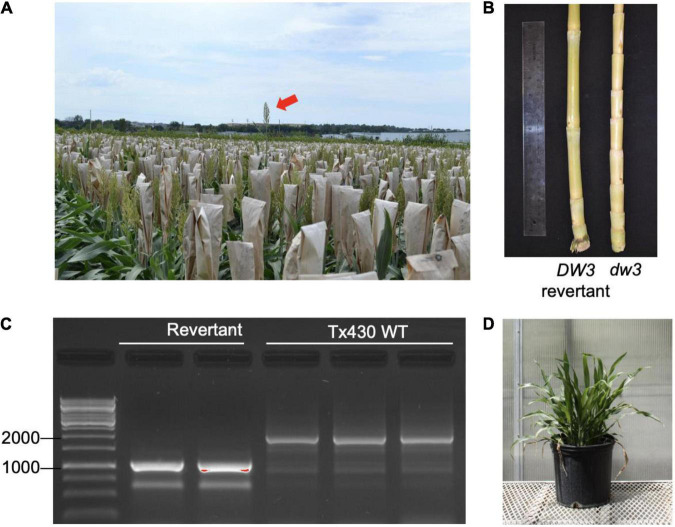
Sorghum *dw3* reversion in Tx430. **(A)** Tx430 plants (*dw3/dw3*) in the field, with visible a *DW3* revertant (red arrow). **(B)** Stalk internode compaction in the TX430-dwarfed *dw3* phenotype (right) compared to a *DW3* revertant (left). **(C)** *DW3* revertants and Tx430 wild-type (*dw3/dw3*) genotypes assayed by PCR. Fragment size (bp) marker lane on left. *Dw3* and *dw3* alleles were discriminated by the presence of a tandem repeat of 882 bp in exon 5; the wild-type plants showed a major band (>1,500 bp). Polymerase chain reaction (PCR) conditions were optimized for amplification of revertant product. **(D)** MSH1-RNAi transgene-null (memory) plant containing the functional *DW3* allele. Under the conditions of this study, *DW3* was not detectable in the phenotype of MSH1-RNAi transgene-null (memory) plants.

Plant height in sorghum is a quantitative trait controlled by four major genes: *Dw1, Dw2, Dw3*, and *Dw4* (Quinby and Karper, 1954). *Dw3*, which encodes an auxin efflux transporter involved in internode elongation (Multani et al., 2003), is a mutant in the Tx430 line used in our study. The *dw3* allele is unstable and can spontaneously revert, producing tall types at a frequency of 0.1–0.5% (Multani et al., 2003). Tx430 contains the *dw3* allele, and we observe evidence of tall revertants in Tx430*_*msh1*_* materials, raising the possibility that *msh1* might increase genome instability or recombination (Figure 1). The *msh1* phenotype is epistatic to plant height in sorghum (Figure 1D), so *dw3* reversion can be masked by *MSH1* suppression and then become evident in progeny following crosses to a wild type. Because loss of function in the unstable *dw3* is caused by 882–base pair direct duplication in exon 5 of the gene, a revertant can be readily identified. These features make *Dw3* a useful marker to investigate genome stability in *msh1*-derived lines.

To ensure against heritable *msh1* background effects, we conducted experiments with *de novo msh1*-modified Tx430 materials that were generated independently from materials used in our previous study (Santamaria et al., 2014). The process involved genotyping each Tx430 wild-type plant, as well as MSH1-RNAi transgene-null memory lines to be used (Figure 1C), to ensure a homozygous *dw3* genotype prior to crossing (Supplementary Figure 1). To generate the MSH1-RNAi transgene-null lines (also denoted as *msh1* memory lines), MSH1-RNAi transgenic plants were confirmed for *MSH1* suppression (Supplementary Figure 1A) and self-pollinated to segregate the transgene (Supplementary Figure 1B). Derived *msh1* memory lines were reciprocally crossed with Tx430 wild-type plants to generate epi-F1 and epi-F2 populations (Supplementary Figure 1B). We investigated *dw3* reversion frequency in Tx430 wild-type, *msh1* memory lines, and derived epi-F1 and -F2 populations under both greenhouse and field conditions. Results showed that reversion frequencies in the *msh1* memory line (0%), epi-F1 (0.5%), and epi-F2 (0.13%) populations did not differ significantly from the Tx430 wild-type (0.150%) or the Tx430 wild type x wild type F1 (0.55%) and F2 (0.07%) (Fisher-exact test, α = 0.05) (Table 1). These results suggest that the *msh1*-associated epigenetic effects in sorghum have little or no detectable effect on the allele instability of this type.

**TABLE 1:** Evaluation of *dw3* reversion frequencies in *msh1*-derived genotypes and isogenic wild-type Tx430.

	Genotype	Total plant tested	Number of revertants	Reversion frequencies	*P*-value
Greenhouse	Epi-F1 (MSH1 *memory* line × Tx430)	602	3	0.50%	0.625
	Tx430 × Tx430 F1 control	547	3	0.55%	0.354
	MSH1 memory line	472	0	0	1.000
Field	Tx430 WT	6,650	10	0.15%	1.000
	Epi-F2 (MSH1 *memory* line × Tx430)	3,876	5	0.13%	1.000
	Tx430 × Tx430 F2 control	4,502	3	0.07%	0.383

*Fisher’s exact test with α = 0.05 is performed to test significance.*

### Enhanced Phenotypic Variation in the Epi-F2 Population

**FIGURE 2:**
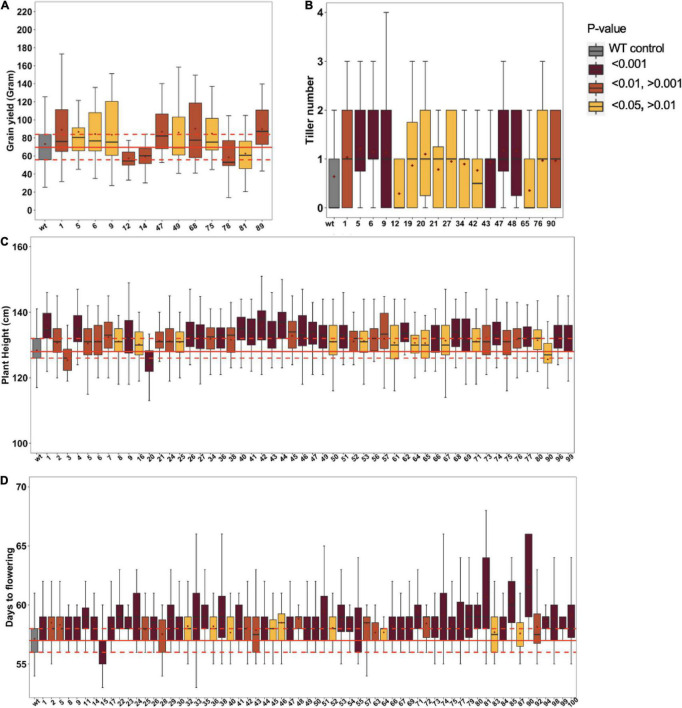
Phenotypic variation in Epi-F2 lines. Boxplot diagrams of epi-F2 lines significantly different from wild-type Tx430 for **(A)** grain yield, where the least squares mean of each line was computed and a linear mixed-effect model was used for pairwise comparisons between each F2 line and the wild type; **(B)** tiller number, where the least squares mean of each line was computed and a Poisson model with log link was used for pairwise comparisons between each F2 line and the wild type; **(C)** plant height, where the least squares mean of each line was computed and a linear mixed-effect model was used to make pairwise comparisons between each F2 line and the wild type; and **(D)** days to flowering, where the least squares mean of each line was computed and a GAMMA model was used to make pairwise comparisons between each F2 line and the wild type. WT data are from 6650 Tx430 WT plants and 4502 Tx430 WT × Tx430 WT F2 plants. We pooled the Tx430 WT and Tx430 WT × Tx430 WT F2 data, since there was no significant difference between the two types of controls. Epi-F2 data are from the progeny derived from multiple individual WT crosses to *msh1* Epi-F1 plants.

The derived epi-F1 plants resembled the wild type in phenotype irrespective of crossing direction, which is consistent with earlier reports (Santamaria et al., 2014). The restored wild-type phenotype in F1 individuals from reciprocal crosses argues against organellar-derived genetic effects, a relevant consideration since MSH1 protein localizes to mitochondria and plastids (Abdelnoor et al., 2003; Xu et al., 2011). To systematically study epi-F2, we conducted field trials to test 100 different epi-F2 families, with at least 45 individuals from each measured for plant height, days to flowering, tillering, and grain yield per individual plant. The *msh1*-derived F2 populations showed increased variation in all traits relative to wild-type checks (Figure 2). For grain yield, 9 families showed significantly higher and 4 significantly lower yield out of the 100 F2 families (Figure 2A). For tillering, 15 lines produced higher and 2 lines lower tiller number (Figure 2B). For plant height, 50 families were significantly taller, and 3 were significantly shorter (Figure 2C), and for days to flowering, 63 were delayed in flowering, and 1 was delayed in early flowering (Figure 2D). We consistently observed this type of skewing in variation distributions. There were no considerable correlations among the traits except for a 52% correlation between tillering and grain yield. The results are particularly notable given that all the observed variation is presumed non-genetic and suggests potential for breeding value.

### Optimal Selection Strategy for Epigenetic Variation

**FIGURE 3:**
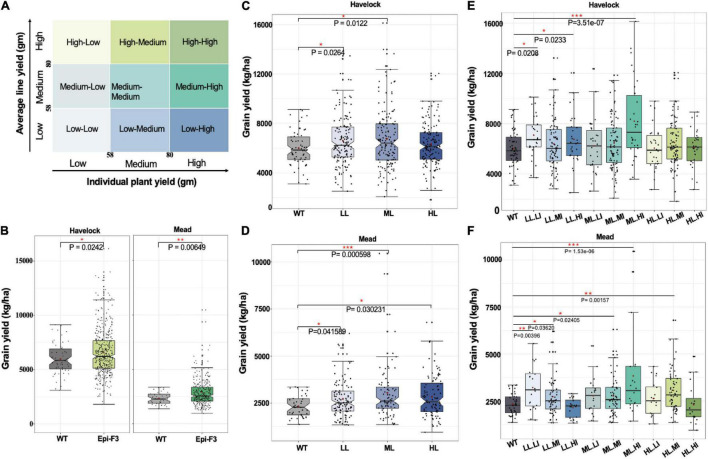
Epi-F3 field testing of selection strategies. **(A)** Grain yield was used as the desired trait in the selection experiment. Each epi F2 line from the field trial from previous year was classified into three groups based on the line yield mean of the 30th and 70th percentiles of wild-type grain yield for low (line yield mean <58), medium (58 ≤ line yield mean ≤ 80), and high (line yield mean >80). The same cutoffs were used to classify each individual plant in every line into three categories (low, medium, and high), with the combination giving nine categories in total. Three putative selection strategies included (1) unselected, (2) selected based on line mean, and (3) selected based on line mean and individual selection, to be tested in the epi-F3 field trial. **(B)** A total of 150 epi-F3 and 19 wild-type lines were tested in the field with a 13 × 13 lattice (incomplete block) design, with 3 replications at Lincoln, NE, and 2 replications at Mead, NE. Grain yield (kg/ha; calculated at 140 g kg^–1^ moisture content) of all 150 epi-F3 lines in each location were compared to WT. **(B)** Shows the outcome of selection strategy 1, unselected. **(C)** Grain yield in kg/ha of all the WTs compared to the epi-F3 groups, with selection based on line mean (LL, low line mean; ML, medium line mean; HL, high line mean) at Havelock. **(D)** Line mean selection results at the Mead location. **(E)** Grain yield (kg/ha) of all the WTs compared to the epi-F3 groups with selection based on line mean and individual selection (LL.LI, low line mean, low individual; LL.MI, low line mean, medium individual; LL.HI, low line mean, high individual; ML.LI, medium line mean, low individual; ML.MI, medium line mean, medium individual; ML.HI, medium line mean, high individual; HL.LI, high line mean, low individual; HL.MI, high line mean, medium individual; HL.HI, high line mean, high individual) at Havelock. **(F)** Line mean and individual selection at Mead, NE. The least squares mean of each line was computed and a linear mixed-effect model was used for pairwise comparisons between each F3 line and the wild type. *p-value < 0.05, **0.001 < *p*-value < 0.01, ***p-value < 0.001.

To learn whether observed epi-F2 phenotypic variation could be selected for incorporation to breeding, we used grain yield as the target trait and designed a field trial to identify the best selection strategy. Epi-F2 families from the previous year’s field trial were classified into three groups based on yield means in the 30th and 70th percentiles of wild-type grain yield, with low (line yield mean <58 g), medium (58 g ≤ line yield mean ≤ 80 g), and high (line yield mean >80 g). The same cutoffs were then used to classify each individual plant within every family to three categories of flow, medium and high, with the combined classifications producing nine categories of low line mean, low individual (LL.LI); low line mean, medium individual (LL.MI); low line mean, high individual (LL.HI), and so on (Figure 3A).

We conducted the experiment in two Nebraska locations, Havelock and Mead, with Havelock conditions approximating optimal soil moisture and fertility (30 ppm nitrogen), and Mead conditions at low soil nitrogen levels (8 ppm) approximating “marginal” field conditions. Our approach was designed to permit the comparison of three selection strategies: unselected, selection based on line mean, and individual selection based on line mean. The outcome with no selection is shown in Figure 3B. Similar to epi-F2, the epi-F3 populations showed greater variation than the wild type for grain yield in both locations (Figure 3B). The mean yield for the epi-F3 populations was 6,565 kg/ha at Havelock and 2,846 kg/ha at Mead, which is significantly higher than wild-type performance, at 5,999 kg/ha and 2,335 kg/ha, respectively. The total yield increase achieved with no selection was 9.4% for the Havelock trial and 21.9% for the Mead trial.

Incorporating selection based on line means identified two groups for the Havelock location, the low line and medium line mean groups, which reached significantly higher yield than the wild-type control. The medium line mean group provided the best effect, with a yield increase of 12.6% (Figure 3C). In the Mead location, all the three groups achieved higher grain yield than the wild-type control, with the medium line mean group again performing best and achieving 30% yield increase compared to the wild type (Figure 3D).

Analysis of data with the “selection based on individual and line mean” strategy identified three groups: medium line-high individual (ML.HI), low line-low individual (LL.LI), and low line-medium individual (LL.MI). These groups were higher-yielding than the control at Havelock, while four groups yielded higher at Mead (Figures 3E,F). The medium line, high individual group appeared to represent the most productive lines among all the nine groups, achieving a 36.7% yield increase compared to the wild type at Havelock and a 64.3% yield increase at Mead (Figure 3D). These data suggest that variation generated epigenetically can provide a significant potential in the field, and that this process can benefit from a well-designed breeding strategy.

### Epi-F3 Line Resilience to Low Nitrogen Stress

**FIGURE 4:**
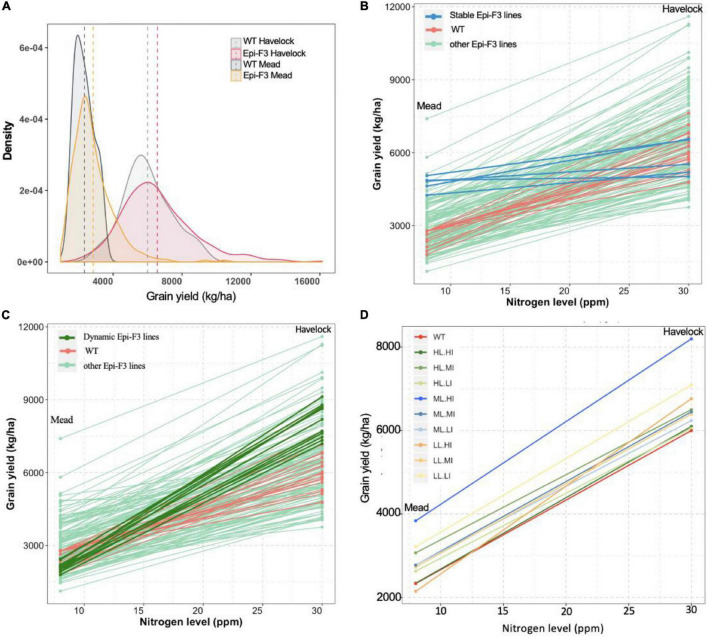
Epi-F3 line resilience in response to low nitrogen was assessed. **(A)** Grain yield density plot of the WT and epi-F3 lines under normal nitrogen (Havelock, 30 ppm of nitrogen) and low nitrogen (Mead, 8 ppm of nitrogen) conditions. **(B,C)** Grain yield for the 150 epi-F3 lines and 19 wild-type lines, with each line in the plot representing one line, in the Havelock and Mead locations. “Stable” epi-F3 lines (epi-line yield differential <30%) are highlighted in blue in **(B)**. “Dynamic” epi-F3 lines (epi-line yield differential >30%) are highlighted in dark green in **(C)**. **(D)** Grain yield differentials of the nine categories for selection based on line mean and individual selection (LL.LI, low line mean, low individual; LL.MI, low line mean, medium individual; LL.HI, low line mean, high individual; and so on). Each line represents a category, and dot represents the average grain yield of all treatments in the given category in the indicated location. For each category, at least 10 lines were included, with three categories having 30 lines (LL.MI, ML.MI, and HL.MI).

The level of epi-F3 line out-performance over the wild type at the Mead location was surprising. The Mead site is used for low nitrogen stress testing, suggesting that the epi-F3 lines have some level of tolerance to soil nutrient stress. In the grain yield density plot, we observed a greater distribution tail toward higher yield in the epi-F3 population, a trend that was more significant in the Mead location. Overall yield differential for the wild-type lines with adequate nitrogen (Havelock, 30 ppm nitrogen) compared to low nitrogen conditions (Mead, 8 ppm nitrogen) was 61.1%, versus 56.6% for the epi-lines (Figure 4A), suggesting that the epi-F3 lines showed slightly overall better resilience to a low-nitrogen environment.

We then looked at response to low nitrogen in the individual epi-F3 lines, observing a considerable variation within the epi-F3 lines for yield loss rate (line slope in Figures 4B,C). With <30% yield differential between normal and low nitrogen as cutoff to define “stable” lines, we identified 5 stable epi-lines (highlighted in blue, Figure 4B), but no wild-type lines met this criterion. We also identified 11 “dynamic” epi-lines; these lines showed a yield differential of >70% as cutoff (highlighted in dark green, Figure 4C). Most of these dynamic epi-F3 lines showed significantly higher yield than the wild type under a normal nitrogen environment but produced yield at wild-type levels under low nitrogen conditions. The dynamic epi-lines (mean grain yield 8,126 kg/ha) showed a 35.5% yield increase over the wild type under normal nitrogen conditions, but suffered greater yield loss (74.1%) under low nitrogen conditions. The 5 stable epi-lines, with a mean grain yield of 5,764 kg/ha, approximated the yield of the wild type (mean grain yield 5,999 kg/ha) under normal nitrogen conditions, but suffered only an 18.1% yield loss when grown under low nitrogen conditions, a significantly smaller decline than the 61.1% seen in the wild type.

The data reflect a trade-off between low nitrogen tolerance and yield, but imply that with correct selection, it is feasible to identify epi-lines that achieve high yield and have low nitrogen tolerance concurrently. The medium line, high individual (ML.HI) group showed higher yield increases at 36.7 and 64.3% than the wild type under normal and low nitrogen conditions, and a more moderate yield differential (53.2%) than the wild type (61.1%) (Figure 4D). These experiments indicate that selection of the ML.HI group provides a distinctive advantage for these environmental conditions and imply that epigenetic plasticity could contribute to resilience in populations.

### Altered Gene Expression in Epi-Lines

To investigate gene expression features of non-genetic variation in sorghum, we performed an RNA-seq analysis to two contrasting sorghum epi-F3 lines that showed distinct growth rates as early as 12 days after germination. We denoted the epi-line with higher growth rate as “epi-F3 high” and the epi-line with lower growth rate as “epi-F3 low.” RNA was extracted from young leaves of 3 individual 24-day-old plants from epi-F3 high and epi-F3 low. This analysis identified 1,331 differentially expressed genes (DEGs) that, with sorghum genome annotation information, produced 9 significantly enriched biological process pathways for response to defense, photosynthesis, and DNA replication processes (Supplementary Figure 4).

**FIGURE 5:**
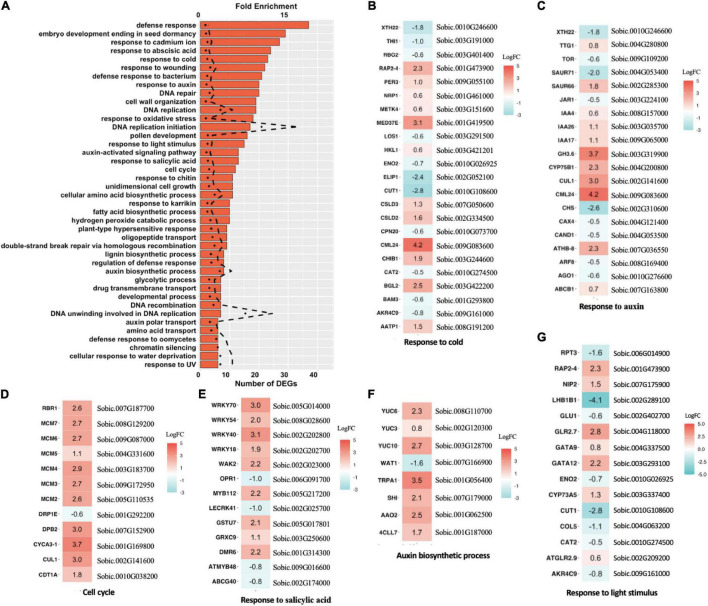
Differentially expressed genes (DEGs) and enriched pathways in epi-F3 lines. **(A)** Significantly enriched Gene Ontology (GO) pathways for DEGs identified in the high-performance epi-F3 vs. low-performance epi-F3 line comparison. Bar graph represents the number of DEGs, and dotted line represents fold enrichment for the enrichment test. One-sided Fisher’s exact test was performed to compute FDR as implemented in DAVID GO, and *p* < 0.05 is the cut-off for significance. **(B–G)** Expression change of individual genes in the significantly enriched pathways for response to cold, response to auxin, cell cycle, response to salicylic acid, auxin biosynthetic process, and response to light stimulus. Sorghum gene ID and corresponding Arabidopsis homolog gene name are listed together. *Sorghum bicolor* NCBIv3.49.gff3 file was used for annotation. NCBI BLASTP was used to identify the homolog of sorghum genes in Arabidopsis (TAIR10 version 38). Genes upregulated in the high-performance line are in red, and downregulated genes are in blue.

We performed Arabidopsis annotation to the DEG data set to identify putative homologous genes in Arabidopsis using a BLASTP algorithm. By Arabidopsis annotation, we obtained a more comprehensive profile of 42 enriched biological process pathways with at least five DEGs each (Figure 5A). DNA replication-related pathways were most significantly enriched, followed by auxin-related pathways, and response to stimulus pathways (Figures 5B–G). The pathway responses appeared consistent with a growth enhancement phenotype; for example, all identified cell cycle pathways were upregulated, including multiple MCM family proteins (Figure 5D). The MCM complex, encoded by *MCM2-7*, plays a vital role in DNA replication (Herridge et al., 2014), implying that the vigor-enhanced epi-line undergoes more robust DNA replication and cell division. The derived epi-line profile also resembled the DEG profile identified for heterosis in Arabidopsis Ler × C24 crosses, where genes responding to salicylic acid and cold show significant change (Wang et al., 2015). The results in sorghum suggest that *msh1*-associated epi-line growth enhancement overlaps with heterosis processes.

To investigate the extent that *msh1* epi-line gene expression profiles may be conserved across species, and whether Arabidopsis serves as an effective model system for crop investigations, we compared the sorghum epi-line profile with previously published Arabidopsis DEG data. Arabidopsis heritable enhancement-through-grafting (HEG) datasets are derived by comparing graft progenies from a Col-0 wild-type scion in *msh1* rootstock (HEG line), which displays robust growth vigor, to a Col-0 wild-type scion in a wild-type rootstock control graft progeny, which shows wild type phenotype (Kundariya et al., 2020). To compare the Arabidopsis and sorghum data sets directly, we identified Arabidopsis homologous genes for sorghum using BLASTP, and Arabidopsis gene IDs were used in gene pathway enrichment analysis. We identified 48 enriched pathways from the Arabidopsis HEG line data set, with 14 that overlapped with sorghum and reflected numerous abiotic and biotic stress response processes (Supplementary Figure 5). The most prominent within these 14 were related to auxin response/regulation and cold response (Figure 5).

In keeping with the identification of response to auxin signals in *msh1*-derived growth enhancement, a prominent Arabidopsis HEG line phenotype is enhanced root growth that is sensitive to auxin inhibitor treatment (Kundariya et al., 2020). To assess sorghum epi-line root effects, we surveyed three different sorghum epi-F3 line root phenotypes selected based on aboveground growth (Supplementary Figure 6B). The sorghum epi-F3 lines varied in primary root length and root dry weight, with two of the three lines surveyed showing significant increase in primary root length and root dry weight over the WT control (Supplementary Figure 6), consistent with non-genetic variation in root phenotype that may be amenable to selection.

**FIGURE 6:**
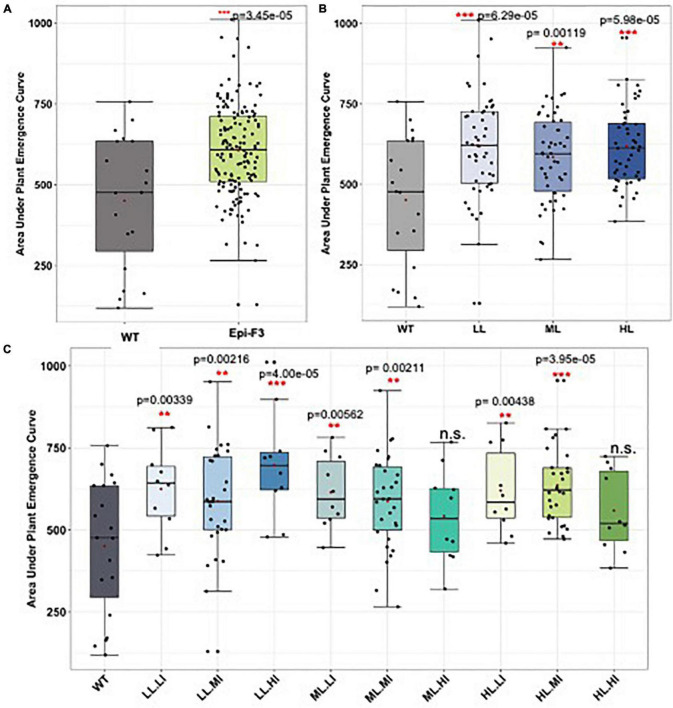
Field cold stress seed germination experiment. A total of 150 epi-F3 and 19 wild-type sorghum Tx430 lines were tested in the field for germination rate under cold stress, with planting at Havelock over two seasons. Planting day soil temperature at 10 cm depth was 11.7°C for experiment 1 and 15.7°C for experiment 2. Standard plantings were carried out at Havelock Farm in mid-May (exp. 1) and mid-June (exp. 2) to assess optimal seed germination rates for each line. Each point represents the average of 2-year data for each line. Plant emergence (number of plants) was recorded on the following days after planting: days 13, 14, 15, 16, 17, 18, 20, 22, 25, 27, 32, 39, and 46. Adjusted emergence data from the early plantings were obtained by dividing early planting emergence by standard planting emergence. Area under the plant emergence curve (AUPEC) and emergence rates were calculated using the adjusted emergence data. Area under the plant emergence curve was calculated as: $\text{AUPEC}\;\text{=}\;\text{Σ[(}PE_{n}\text{+}PE_{\left(n+1\right)}\text{)/2]*}Y_{\left(n+1\right)}-Y_{n^{\prime }}$ Where PE_*n*_ = plant emergence at time *n*, and *Y*_*n*_ = day at time *n*. **(A)** Area under the plant emergence curve of all the 150 epi-F3 lines under cold stress were compared to the WT. **(B)** Area under the plant emergence curve of the WT compared to the epi-F3 groups, where epi-F3 populations are separated based on the line mean selection strategy used in the yield study. **(C)** Area under the plant emergence curve of the WT compared to the epi-F3 groups, where epi-F3 populations are separated based on the line mean and individual selection strategy used in the yield study. The least squares mean of each line was computed and a linear mixed-effect model was used for pairwise comparisons between each F3 line and the wild type. ^**^0.001 < *p*-value < 0.01, and ^***^p-value < 0.001.

Arabidopsis *msh1*-derived variation has also been documented for cold tolerance (Raju et al., 2018b). Consequently, we evaluated sorghum non-genetic variation for cold response during seed germination and in plant growth within 2 years of field trials. Figure 6 shows evidence of enhanced variation in seed germination under cold soil conditions in sorghum epi-F3 lines relative to the wild type. Following germination, we also observed a variation in sorghum early plant growth under cold conditions (Supplementary Figure 7 and Supplementary Table 6), with evidence of increased plant height in young epi-F3 plants relative to the wild type following germination under cold conditions. These observations indicate that Arabidopsis can serve as an effective model for predicting crop *msh1*-derived phenotypic variation and raise the possibility of identifying signature pathways and individual markers of relevance for *msh1*-derived growth vigor phenotypes.

### Comparison of the *msh1* Effects With Genetic Selection Outcomes for Tolerance to Low Nitrogen Conditions

A previous study on genetic variation in sorghum for tolerance to low nitrogen field conditions developed gene expression profiles of 3 lines sensitive to low nitrogen (CK60, BTX623, and a low NUE RIL line) and 4 tolerant lines (China 17, San Chi San, KS78, and a high NUE RIL line), providing evidence of differential gene expression and an indication of genetic potential for low nitrogen stress tolerance in sorghum germplasm (Gelli et al., 2014). We analyzed these previously developed data in comparison to epigenetic variation observed in the singular line, Tx430, exposed to very similar field conditions for nitrogen depletion in this study. For this analysis, we included data from Tx430 high performance versus low performance epi-lines to investigate the relationship between epigenetic and genetic variations.

Although DEG data from the earlier genetic variation study was derived from root tissues, while DEG data from the Tx430 epi-line comparisons were prepared from leaf tissues, and the genetic contrast data set of CK60 (sensitive) versus high NUE RIL (tolerant) shared 130 genes common with the Tx430 epi-line contrast, accounting for 20% of the genetic and 13.7% of the epi-line data sets (Supplementary Figure 8A). Many of the identified common DEGs between the two contrasted data sets appeared relevant to nitrogen-related responses. For example, the sorghum gene *SORBI_3003G191000* is homologous to Arabidopsis *THI1* (AT5G54770), a thiamine biosynthetic gene that functions in thiamine biosynthesis and mitochondrial DNA damage tolerance. *SORBI_3002G272700* (Arabidopsis homolog *CYP78A9*, AT3G61880), encodes a cytochrome p450 monooxygenase that, upon overexpression, allows parthenocarpic fruit growth. *SORBI_3010G172200* (Arabidopsis homolog, AT4G39950, *CYP79B2*), is a cytochrome p450 gene that participates in tryptophan metabolism, and converts Trp to indole-3-acetaldoxime (IAOx), a precursor to IAA. *SORBI_3006G249400 (GLN2), SORBI_3001G248500 (GR2)*, and *SORBI_3005G017801 (GSTU7)* are all involved in glutathione biosynthesis and transduction.

However, comparison of the enriched pathways identified by the overlap of genetic and epigenetic DEG data sets revealed 18 shared pathways, most identified by the sorghum-Arabidopsis comparisons of *msh1* epi-line effects, including response to auxin, cell wall organization, lignin biosynthetic process, and oligopeptide transport (Supplementary Figures 5, 8B). We take these observations to imply that gene pathways influenced by genetic selection for low nitrogen tolerance and related to pathways in *msh1* reprogramming are likely those that contribute to phenotypic plasticity and resilience.

The previous study on genetic variation in sorghum also evaluated the low N-sensitive (CK60, BTX623, and low NUE RIL line) and 4 low N-tolerant lines (China 17, San chi San, KS78, and high NUE RIL line) for performance in the field (Gelli et al., 2014). Yield losses under low nitrogen stress, relative to optimal cultivation, amounted to 62.1, 57.1, and 54.8% for the 3 low N-sensitive lines, and 29.1, 21.9, 46.3, and 13% for the 4 tolerant lines (Gelli et al., 2014). Our current observation of 5 Tx430 epi-lines suffering less than 18.1% yield loss under low nitrogen stress suggests that the phenotypic plasticity that arises with *msh1* manipulation may be comparable in overall effect to that achieved through genetic selection.

## Discussion

The concept of selecting epigenetic phenotype variation in crops has been proposed but not implemented on a field scale for practical crop improvement. Sorghum presents an excellent system for investigating enhanced phenotypic plasticity because of its inherent drought tolerance, broad utility for grain, sugar and biomass, and its facility for cultivation in annual or perennial mode (Cox et al., 2018). Sorghum originated in tropical regions and is a cold- and frost-sensitive plant. Low-temperature stresses, such as chilling and frost, greatly affect the germination and growth of the plant, and limit the geographical distribution of the crop. This poses a major problem in temperate environments when sorghum is planted early and cool air and soil temperatures result in poor seedling establishment because of slow emergence rate, reduced emergence percentage, and reduced growth rate after emergence (Singh, 1985). Early planting of sorghum hybrids in temperate regions provides numerous advantages but requires genetic variation to facilitate germination and seedling tolerance to low soil temperatures. The incorporation of strategies for increased cold tolerance would help to expand and stabilize sorghum production under these conditions.

Non-genetic variation is desirable for population survival under rapidly changing environmental conditions (O’Dea et al., 2016; Varotto et al., 2020). Our data suggest that epigenetic variation, if selected correctly, can help mitigate yield loss under adverse field conditions by reducing epitype x environment interaction. Because we were able, in an earlier study, to observe phenotypic variation in sorghum following *MSH1* suppression and memory line development, this study was designed to exploit the potential of this uncommon variation to test alternative selection strategies and implementation for crop improvement. The experimental design used in the study allowed for the retroactive comparison of different selection approaches and outcomes. Surprisingly, yield gain was most significant in the subsequent generation when medium mean lines were selected and with selection of the highest performing individuals from this mid-range group. These data suggest that the highest performing epi-F2 group was slightly diminished in the subsequent generation and argues the benefit of selecting a performance range rather than a stringent upper percentile. If this phenomenon holds true in additional selection cycles, we may begin to define a selection strategy for epigenetic variation. Such a strategy would not only shorten breeding cycles, but would also allow adaptation for larger target cultivation regions.

Our investigations showed that the non-genetic variation arising from *msh1* suppression can afford as much as 37% yield increase in the Tx430 genetic background when grown under favorable field conditions, and as much as 64% increase under nitrogen-limiting conditions. These data imply that inherent phenotypic variation within a genotype may be considerable and can impact GxE buffering. What is not yet known is how this non-genetic phenotypic variation may vary on diverse genetic backgrounds or within a hybrid breeding program. The significant observed GxE variation among genetically identical Tx430 epilines supports the hypothesis that innate stochasticity in plants thought to contribute to bet hedging in natural populations (Simons, 2011) might be detectable and accessible through *msh1* reprogramming (Mackenzie and Kundariya, 2020).

A transcriptome analysis on sorghum was facilitated by comparisons with Arabidopsis data sets, identifying several commonalities in *msh1*-modified gene pathways putatively associated with growth vigor. Two of the identified pathways related to auxin and cold responses were particularly prominent in both Arabidopsis and sorghum and were, thus, pursued as potentially predictive of plant epi-phenotypes. Data supporting *msh1*-derived variation in sorghum root growth and cold temperature seed germination are compelling outcomes of this comparative analysis approach, again pointing to inherent phenotypic variation within the individual genotype that is generally not accessed through traditional breeding. In this case, multispecies analysis served to narrow focus to target processes, a useful method for dissecting complex growth phenotypes to identifiable components.

Although the evaluation of root growth was conducted on only a limited population size of selected sorghum lines, the variation observed in seedling root growth was consistent with what previously observed in Arabidopsis for *msh1*-derived growth vigor in graft progeny (Kundariya et al., 2020). These outcomes argue for potential efficacy of a seedling assay to predict *msh1*-derived vigor. Similarly, previous evidence of *msh1*-derived cold tolerance in Arabidopsis (Raju et al., 2018b), together with sorghum transcriptome data analysis, served to predict variation for cold tolerance in the derived sorghum lines. Tests showed a significantly greater variation and potential for cold tolerance in the *msh1*-derived epi-lines than those of their isogenic wild-type counterparts for seed germination and early plant growth features with no change in anthesis time. Successful early planting of sorghum, requiring cold tolerant seed germination, provides opportunity for crop expansion to new growing areas and efficient resource utilization (Emendack et al., 2021).

Comparison of sorghum genetic to epigenetic variation for response to low nitrogen stress provided evidence that epigenetic variation may be modulating many of the same pathways that comprise genetic selection schemes. The impacts of epigenetic effects were comparable to reduced GxE emanating from genetic selection, supporting the utility of integrating both genetic and epigenetic variations to future breeding efforts for resilience. Our study suggests that the phenotypic plasticity in *msh1*-altered sorghum lines can contribute to a novel strategy for expediting crop environmental resilience.

## Experimental Procedures

### Plant Material and Growth Conditions

The sorghum MSH1-RNAi transgenic lines were developed as described in Xu et al. (2012) and Santamaria et al. (2014). MSH1 suppression was achieved by introduction of an RNAi transgene to inbred line Tx430 *via Agrobacterium* transformation. For genotyping, forward primer 5′-AGGATGTGCTGCAAGGCGATTAAGTTG-3′ and reverse primer 5′-GGTTGA GGAGCCTGAATCTCTGAAGAAC-3′ were used under the following PCR conditions: 5 min of initial denaturation at 95°C, 34 cycles of 30-s denaturation at 95°C, 40 s annealing at 60°C, 2 min extension at 72°C, and 10 min of the final extension at 72°C. In the greenhouse, all sorghum materials were grown at 14-h day length with 28°C/22°C day-night temperatures.

### Field Experimental Design and Data Collection

A total of 100 epi-F2 families (descendants of 100 individual F1 plants) were grown during the summer under rainfed conditions at the University of Nebraska Havelock experiment station in Lincoln, Nebraska (40° 51′ N, 96° 35′ W). An augmented lattice design (incomplete block experimental designs) with three replications was used. Wild-type Tx430 was randomly inserted in each incomplete block to make a 10 × 11 rectangle. In this design, incomplete blocks were grouped into larger blocks and large blocks formed a complete block design, which was taken as a replication. Each individual epi-F2 family appeared once in each replication. A high seeding rate of 7 seeds per foot was used. Excess plants were removed 2 weeks after emergence to make a uniform plant stand with individual plants spaced at 6 in within a row. Phenotypic data were collected from 15 uniformly spaced plants within the central portion of each row including days to flowering (recorded as emergence of the head from the flag leaf sheath), plant height (measured from ground to panicle tip), tillering, and grain yield per individual plant. The main panicles from individual plants were bagged at flowering for self-pollination. The bagged panicles were harvested and threshed individually. Panicles from tillers were not bagged, but their grain weights were included for total grain yield.

To test the optimal selection strategy of an *msh1*-derived epigenetic breeding system, we used grain yield as the focus trait. Based on the 30th and 70th percentiles of wild-type grain yield in the epi-F2 field trial, we classified every epi-F2 line into three groups based on line yield mean, low (line yield mean <58), medium (58 ≤ line yield mean ≤ 80), and high (line yield mean >80). The same cutoffs were used to classify each individual plant in every line into three categories (low, medium, and high), and the combination gave rise to 9 different categories in total. For each category, at least 10 lines were included, with three categories, for 30 lines (low line mean, medium individual group, medium line mean, medium individual group and high line mean, and medium individual group).

With this design, three putative selection strategies included (1) unselected, (2) selected based on line mean, and (3) selected based on line mean and individual selection. A group of 150 epi-F3 and 19 wild-type lines were grown in the field with a 13 × 13 lattice (incomplete block) design, with 3 replications at Havelock (on the regular fertilization program to provide sufficient nitrogen, with nitrogen level approximated at 30 ppm), and 2 replications at Mead, NE, where fertilizer has not been applied for more than 15 years (nitrogen level is approximately 5–8 ppm). Data recorded for each plot included number of days to anthesis, plant height at harvest, grain yield, panicle grain weight, panicle stover weight, seed number, seed weight, test weight, number of grain-bearing panicles, and number of plants. These data were used to calculate grain yield (kg/ha) at 140 g kg^–1^ moisture, threshing percentage, seeds per square meter, seeds per panicle, and panicles per plant.

### Field Data Statistical Analysis

Statistical analysis for plant yield and height in field data involved linear mixed model yij ∼ genotypei + blockj + eij, where genotype i was treated as a fixed effect and block j was treated as a random effect, and eij was the residual error, implemented by lmerTest R package (version 3.1-2). Tests for significant differences in line means were performed by linear mixed hypothesis tests with the lmer function from the same package. Statistical analysis for tiller number and days to flower used a similar approach except the models used were Poisson and GAMMA, respectively.

### Field Cold Stress Germination Experiments

The cold stress germination field testing was also conducted at Havelock Farm. The 150 epi-F3 and 19 Tx430 wild-type lines were tested in the field for germination rate, with early planting in year 1 (experiment 1) on April 20 and in year 2 (experiment 2) on April 16, to introduce cold stress. The planting day soil temperature at 10 cm depth was 11.7 (exp. 1) and 15.7°C (exp 2). Standard planting was then conducted on May 23 (exp. 1) and June 11 (exp. 2) in the same location to measure the seed germination rate of each line and control for seed quality. Each point represents the average of 2-year data for each line. Plant emergence, as number of plants, was recorded after planting on days 13, 14, 15, 16, 17, 18, 20, 22, 25, 27, 32, 39, and 46. Adjusted emergence data were obtained by dividing early planting emergence by standard planting emergence. Area under the plant emergence curve (AUPEC) and emergence rates were calculated with the adjusted emergence data. Area under the plant emergence curve was calculated as:


(1) $$AUPEC=\Sigma {\left[\left(PE_{n}+PE_{\left(n+1\right)}\right)/2\right]}^{*}Y_{\left(n+1\right)}-Y_{n^{\prime },}$$

With *PE*_*n*_ = plant emergence at time *n* and *Y*_*n*_ = day at time *n*.

### Screening for *Dw3* Reversion

Polymerase chain reaction (PCR)-based genotyping was performed to assay for presence or absence of *dw3* mutation (tandem repeat of 882 bp on exon 5) with forward primer 5′-CGTCCTGCAGAAGATGTTCATGAAGG-3′ and reverse primer 5′-GTGCGCCACCACGATGGTGGTGC-3. The Promega GoTaq Green Master Mix, 2X, was used for a 25-μl total volume. The PCR assay was adapted from Farfan Barrero et al. (2012). All the plants used in the *msh1* memory line x Tx430 crosses were screened to confirm the absence of the *DW3* allele that conditions a tall phenotype relative to Tx430. The F1 progeny was similarly screened.

### RNA Sequencing and Analysis

Sorghum materials were grown in the greenhouse at 28°C (day)/22°C (night) and 14-h day length. Young leaf tissues from 3 individual 4-week-old high-performance and 3 individual low-performance epi-line plants were collected, fast frozen in liquid nitrogen, and stored at −80°C. Leaves were ground in liquid nitrogen and processed for RNA extraction with NucleoSpin RNA Plant Kit (Macherey-Nagel, Düren, Germany). Total RNA-derived libraries were constructed as described in the TruSeq RNA sample preparation v2 Guide and sequenced on the DNBseq 500 platform (BGI-Tech, China), with 150-bp read, paired-end read option, for minimum 60 M reads per sample. Raw sequencing reads were quality-controlled and trimmed with TrimGalore! (version 0.4.1). Trimmed reads were aligned to the *Sorghum bicolor* reference genome (NCBIv3) using STAR (version 2.7.3a) with –twopassMode = Basic and –outFilterMultimapNmax = 1 parameter, retaining only uniquely mapped reads. The read count data were generated from BAM files with the QoRTs software package (version v1.3.0) at –minMAPQ = 25 option. The edgeR package (version 3.26.8) was used for gene count normalization and identification of DEGs (adj. *p*Val ≤ 0.1, | log2FC| ≥ 0.5). DAVID Bioinformatics Resources 6.8 was used for GO function enrichment analysis (Huang et al., 2009). GO terms with Fisher’s exact *P*-value ≤ 0.1 and gene counts ≥ 5 in each GO were selected. The *Sorghum bicolor* NCBIv3.49.gff3 file was used for annotation. NCBI BLASTP was used to identify sorghum gene Arabidopsis homologs (TAIR10 version 38), and E score >100 was used as the cutoff. Detailed code is available at https://github.com/genomaths/genomaths.github.io/tree/master/blastp.

### Root Assay

To eliminate the contribution of variation in seed germination, seeds were germinated on wet paper towels. Seeds with similar germination time were then transferred to deep pots (24-cm length and 6-cm diameter) filled with fine sand (∼20 cm) topped with potting mix (∼2 cm) to allow seedlings to root properly. After 12 days, the roots were washed thoroughly with water to remove soil. Primary root length and aboveground height were then measured. To obtain the dry weight of root and aboveground parts, the plants were dried in the greenhouse for 2 weeks before weighing.

## Data Availability Statement

The datasets presented in this study can be found in online repositories. The names of the repository/repositories and accession number(s) can be found in the article/Supplementary Material.

## Author Contributions

DK carried out all the sorghum field, PCR-based experiments, and data analysis. XY conducted RNA seq analysis and prepared the draft of the initial manuscript. RS planned the experimental design and conducted statistical analysis. HK and JR participated in plant phenotype and data analysis. ID participated in greenhouse and field experimental design. SM obtained funding, designed the experiments, participated in data analysis, and prepared the manuscript. All authors contributed to the article and approved the submitted version.

## Conflict of Interest

SM was a co-founder for EpiCrop Technologies Inc., a small startup company that tests the potential value of the MSH1 system in epigenetic crop breeding. The remaining authors declare that the research was conducted in the absence of any commercial or financial relationships that could be construed as a potential conflict of interest.

## Publisher’s Note

All claims expressed in this article are solely those of the authors and do not necessarily represent those of their affiliated organizations, or those of the publisher, the editors and the reviewers. Any product that may be evaluated in this article, or claim that may be made by its manufacturer, is not guaranteed or endorsed by the publisher.

## Supplementary Material

Supplementary Figure 1 Deriving epi-line materials in this study. **(A)** qRT-PCR analysis results for *MSH1* transcript levels in variant phenotype plants with (+) and without (null) *MSH1*-RNAi transgene relative to wildtype Tx430. **(B)** Schematic diagram of the procedure for *MSH1* suppression and *de novo* epi-line development for this study.

Supplementary Figure 2 Plant height density plot for the Havelock summer field trial. The dashed red vertical line represents the mean of each population. The plant height data for 100 epi-F2 lines were pooled make this plot. Likewise, wildtype samples from all incomplete blocks were pooled to construct the density plot. WT represents the data from 6650 Tx430 WT plants plus 4502 Tx430 WT crossed to Tx430 WT F2 plants. We pooled the Tx430 WT and Tx430 WT × Tx430 WT F2 data since there was no significant difference between these two types of controls. Epi-F2 represents progeny from multiple individual WT crosses to *msh1* Epi-F1 plants.

Supplementary Figure 3 Boxplot data for grain yield in epi-F2 families (100 lines shown separately) and the Tx430 wild type in a Havelock summer trial. The horizontal red solid line represents the wildtype median, while top and bottom red dashed lines represent the 75 and 25% wildtype yield percentiles, respectively. The boxplots show the range of observed sample variation. Wildtype samples from all incomplete blocks were pooled for the WT box blot and to compute the median. WT represents data from 6650 Tx430 WT plants and 4502 Tx430 WT × Tx430 WT F2 plants; we pooled the Tx430 WT and Tx430 WT × Tx430 WT F2 data because there was no significant difference between these two controls. Epi-F2 data derive from progeny of multiple individual WT crossed to *msh1* epi-F1 plants.

Supplementary Figure 4 Enriched pathways identified in epi-F3 lines using sorghum genome annotation. Significant enriched GO pathways for DEGs identified in the high-performance epi-F3 line vs. low-performance epi-F3 line comparison. Bar graph represents number of DEGs and dotted line represents fold enrichment. AgriGO v2.0 (Tian et al., 2017) was used for the enrichment test, with *Sorghum bicolor* as the species and default for other parameters.

Supplementary Figure 5 Venn diagram showing enriched pathways identified for sorghum DEGs (epi-F3 line, high-epi vs. low-epi) and Arabidopsis HEG line DEGs. The Arabidopsis heritable enhancement-through-grafting (HEG) DEG dataset was obtained from a previous study (Kundariya et al., 2020). To compare these two species directly, we identified the Arabidopsis homologous genes for sorghum using BLASTP, then conducted GO enrichment analysis. DAVID Bioinformatics Resources 6.8 was used for GO functional enrichment analysis. GO terms with Fisher’s exact *P*-value ≤ 0.1 and gene counts ≥5 in each GO were selected.

Supplementary Figure 6 Root phenotype of epi-F3 compared to WT control. **(A)** The root phenotype of individual plants from different selected epi-F3 lines. **(B)** Above ground phenotype of the selected epi-F3 lines compared to WT. For **(A,B)**, pictures were taken 12 days after germination. **(C)** Primary root length, **(D)** root dry weight, **(E)** plant height, and **(F)** aboveground dry weight of the epi-F3 lines compared to WT control. The Mann–Whitney *U*-test with two-sided alternative hypothesis was used to test significance of the difference of mean in each comparison. Significance codes: **p* < 0.05, ^**^*p* < 0.01, ^***^*p* < 0.001. Data are represented as boxplots, where the middle line is the median, the lower and upper hinges correspond to the first and third quartiles, the upper whisker extends from the hinge to the largest value and the lower whisker extends from the hinge to the smallest value, while data beyond the end of the whiskers are outlying points that are plotted individually, sample size *n* > 10. WT and epi-F3 seeds were sown and geminated on paper towels and transferred to growth pots with fine sand soil; 12-day-old plants were then washed with running water to clean the root and processed for root phenotype parameter measurements. EpiF3-68 and EpiF3-15 were the lines also used in the RNA-seq experiments (Figure 5).

Supplementary Figure 7 Field cold stress germination experiment. 150 epi-F3 and 19 wild type lines were tested in the field for germination rate under cold stress conditions, with early planting at the Havelock site on April 20 (Experiment 1) and April 16 (Experiment 2). Planting day soil temperature at 10 cm depth was 11.7°C (Exp 1) and 15.7°C (Exp 2). Standard planting was carried out on May 23 (Exp. 1) and June 11 (Exp. 2) to obtain seed germination rates for each line. Each point represents the average of 2 year’s data for each line. **(A)** Adjusted seedling emergence rate under cold stress. Final plant emergence was documented 46 days after planting. Adjusted emergence rate was obtained by dividing the early planting emergence by standard planting emergence. **(B)** Plant height and **(C)** days to anthesis for WT and epi-F3 lines under cold and standard conditions. The least squares mean of each line was computed and linear mixed-effect model used for pairwise comparisons between each F3 line and wild type. **p*-value < 0.05, ^**^0.001 < *p*-value < 0.01, ^***^*p*-value < 0.001.

Supplementary Figure 8 Identified overlap between DEGs from the low-N sensitive genotype (CK60) vs. tolerant genotype (High RIL) comparison under low-N condition and the DEG dataset from Tx430 epi-F3 line comparison generated in the study (Supplementary Figure 6). **(A)** Venn diagram of the 130 genes present in both DEG datasets, accounting for 20% of CK60 vs. High RIL DEGs and 13.7% of Tx430 high-epiF3 vs. low-epiF3 DEGs. **(B)** Enriched pathways identified for DEG dataset from the genetic (CK60 vs. High RIL) comparison (Gelli et al., 2014) and the Tx430 epi-line comparison. The genetic comparison DEG dataset was acquired from a previous study (Gelli et al., 2014). To conduct the GO analysis in a more informative way, we identified Arabidopsis homologous genes for both sorghum datasets using BLASTP, then conducted GO enrichment analysis. DAVID Bioinformatics Resource 6.8 was used for GO function enrichment analysis. GO terms with Fisher’s exact *P*-value ≤ 0.1 and gene counts ≥5 in each GO were selected.
