# Supplementary material for: Implementation of Epigenetic Variation in Sorghum Selection and Implications for Crop Resilience Breeding
Source: Front Plant Sci. 2022 Jan 27;12:798243. doi: 10.3389/fpls.2021.798243 (PMC8828589; doi:10.3389/fpls.2021.798243)

Supporting figure 1

a

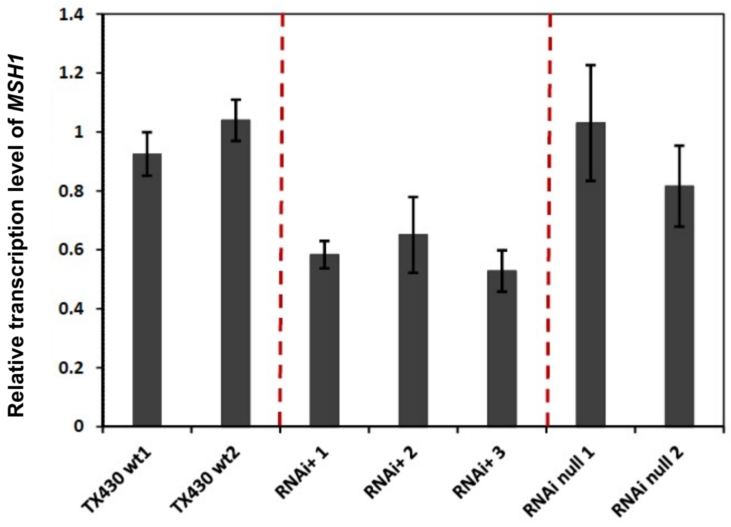

b

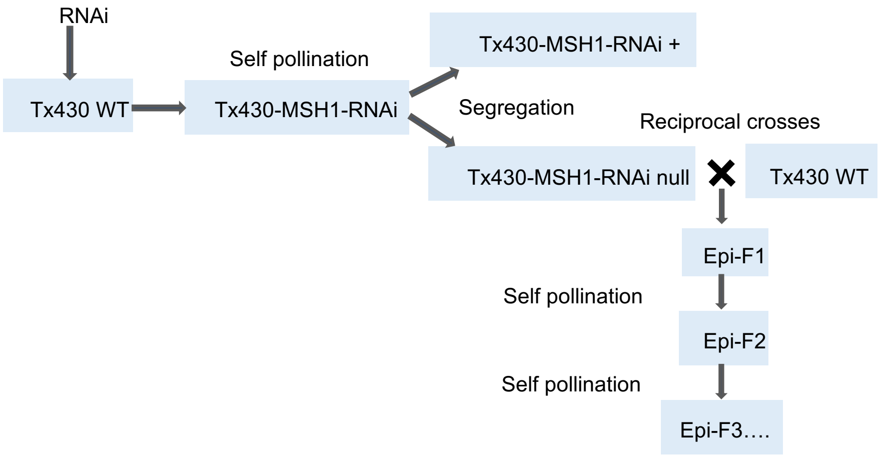

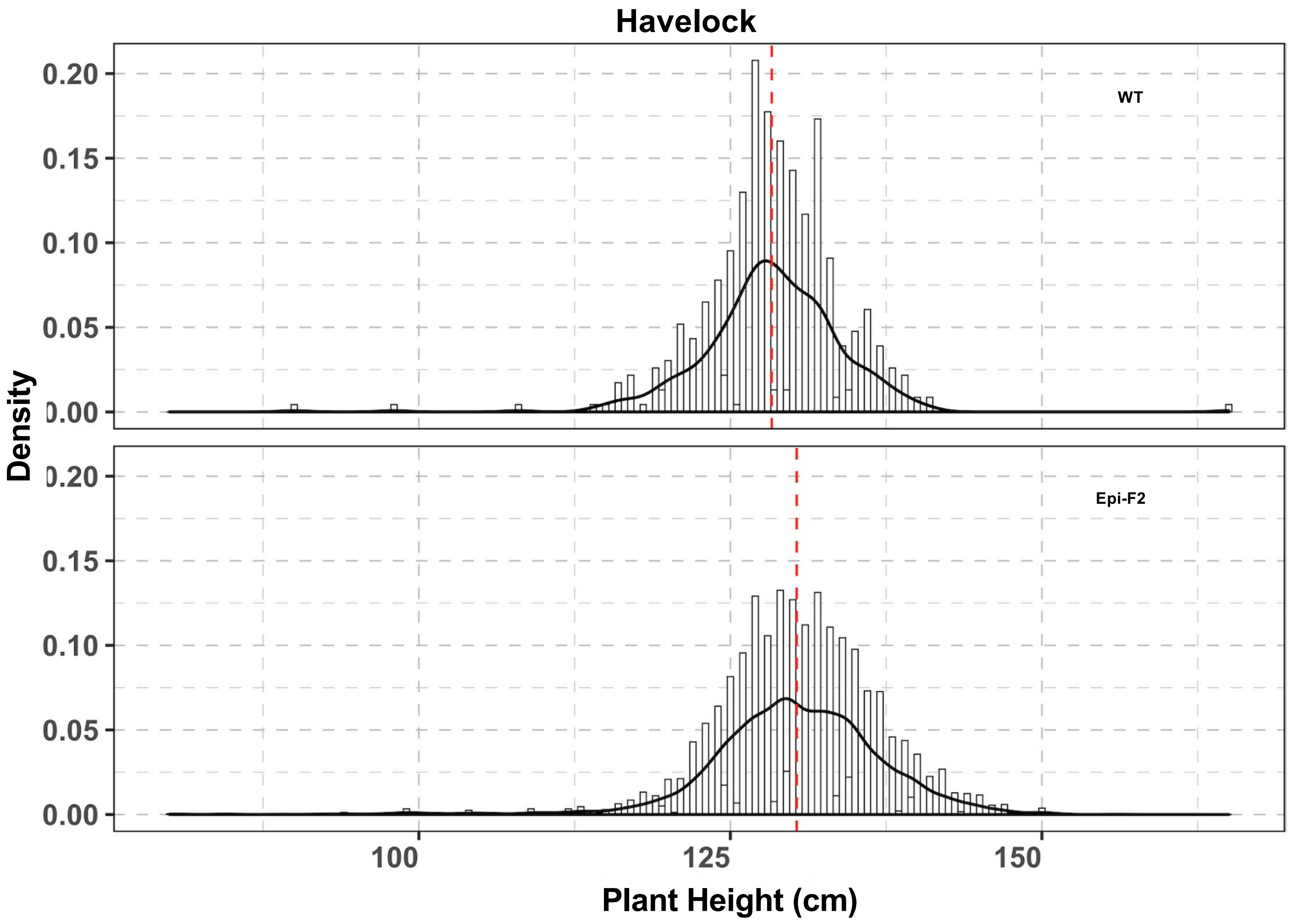

Havelock
WT
Epi-F2
Density
0.20
0.15
0.10
0.05
0.00
100
125
150
Plant Height (cm)

Supporting figure 3

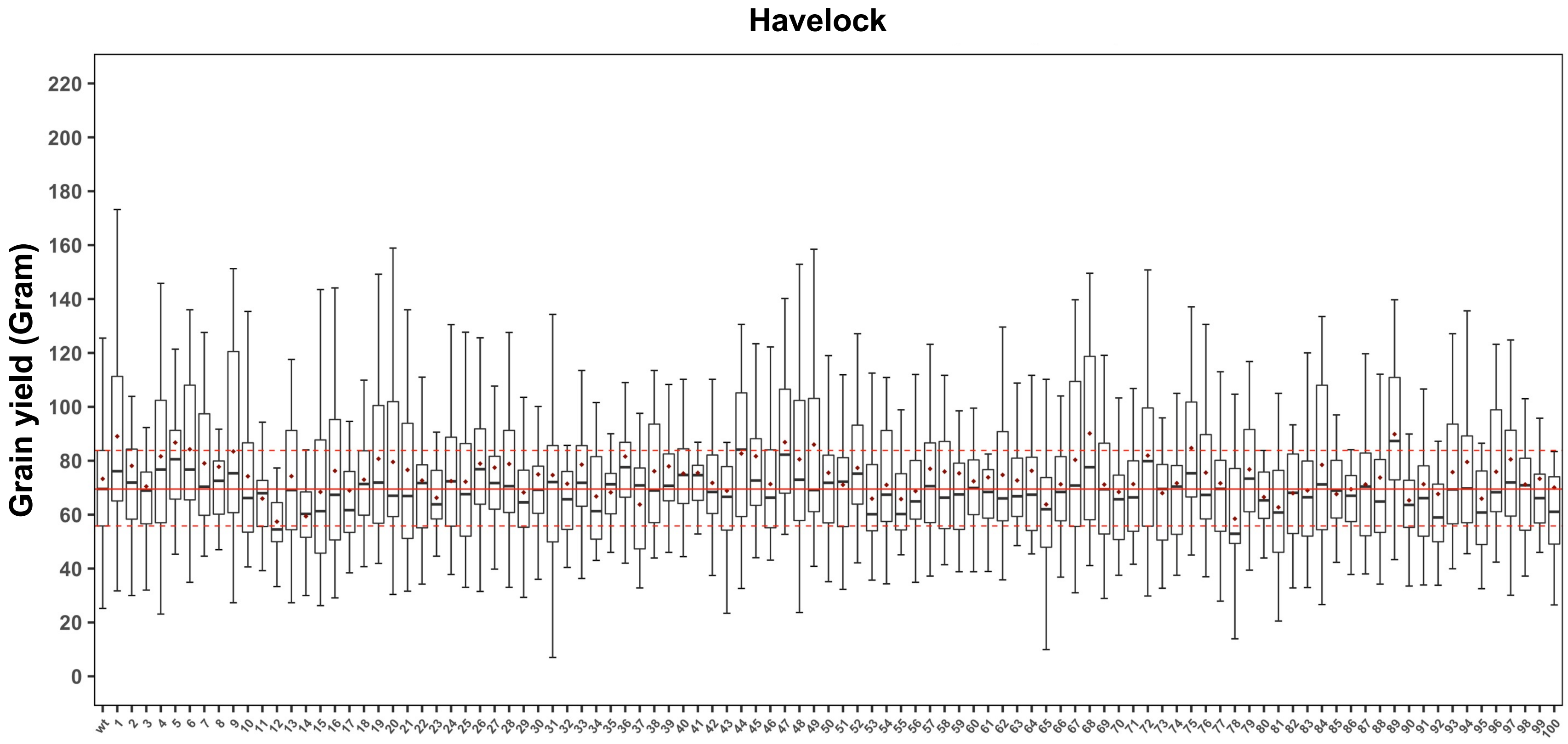

Supporting figure 4

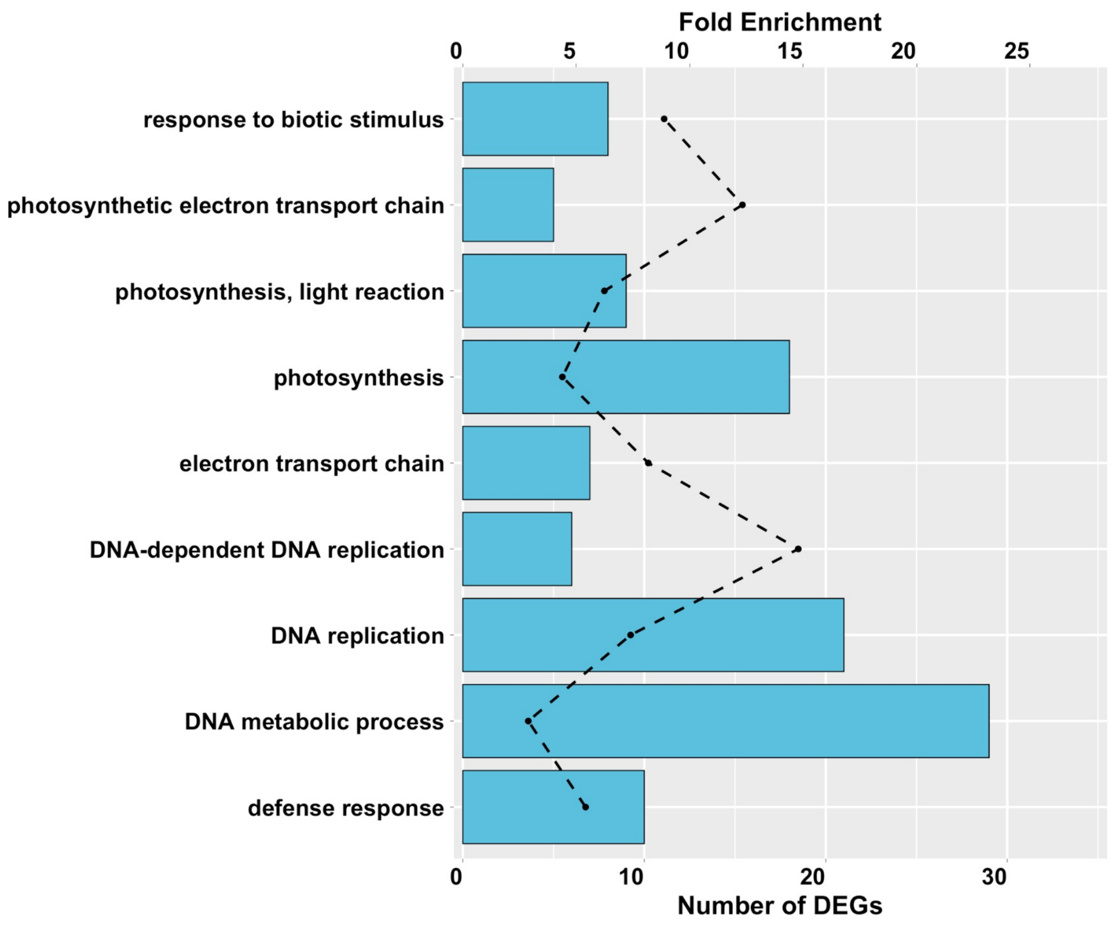

Supporting figure 5

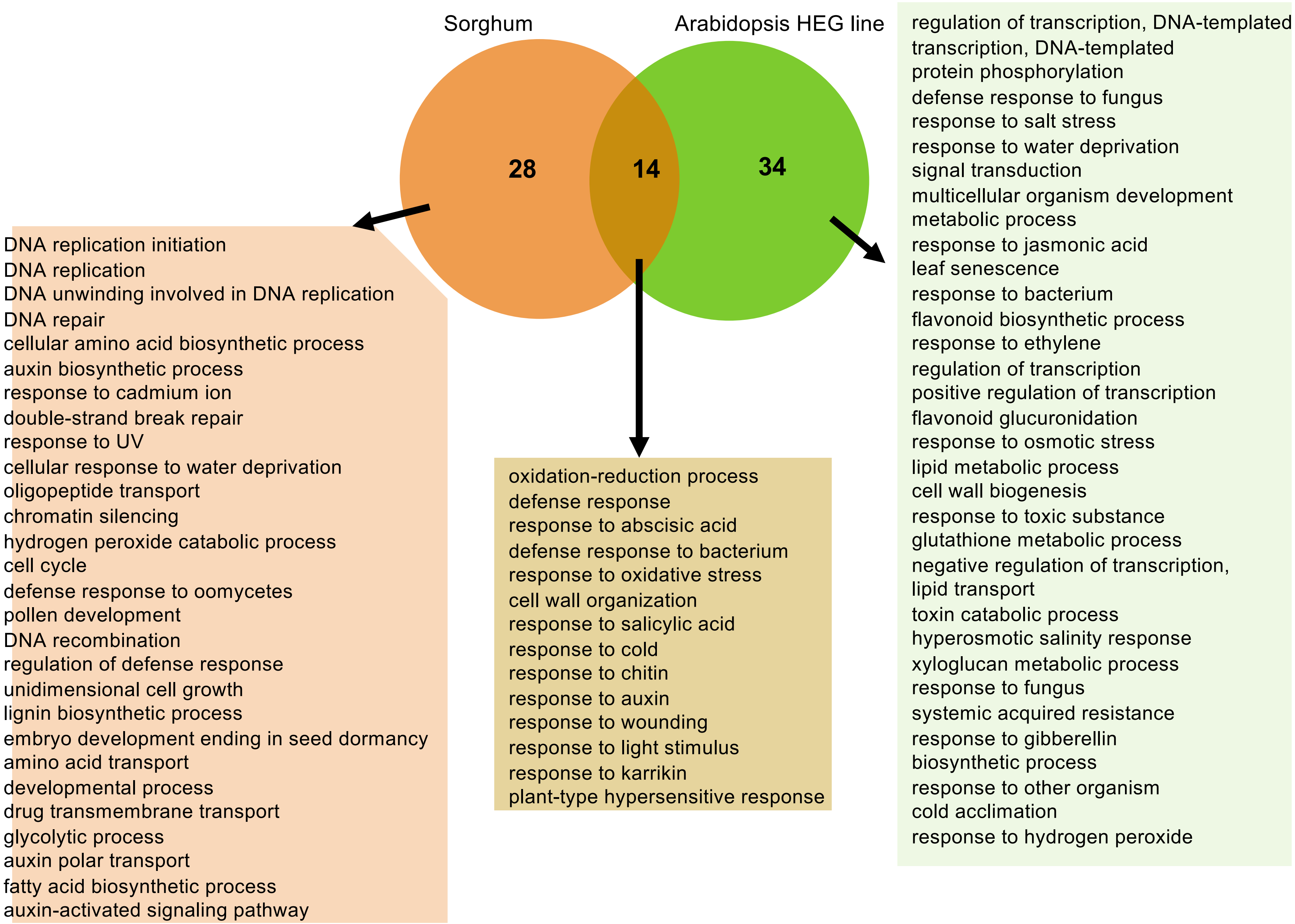

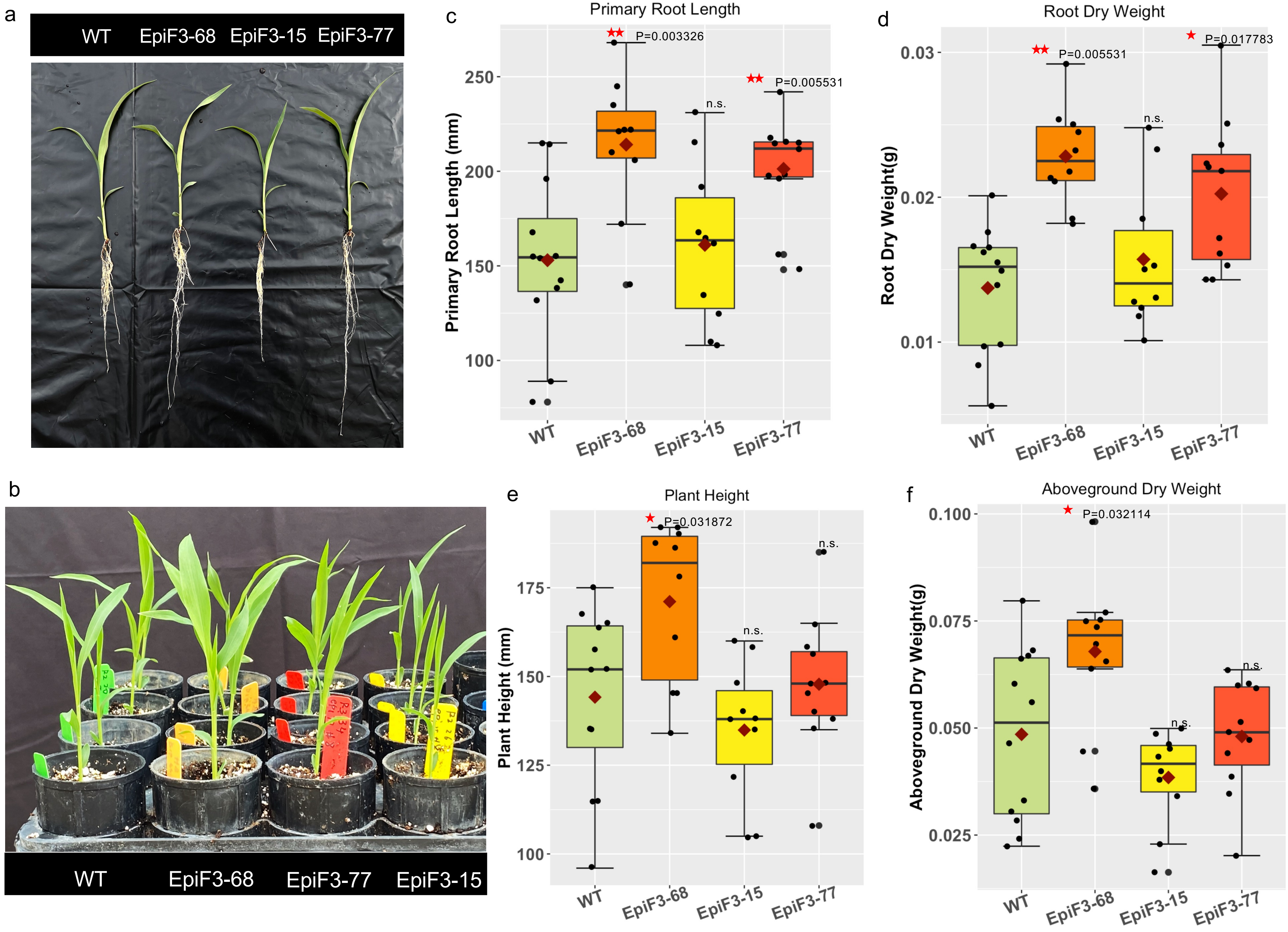

a
WT
EpiF3-68
EpiF3-15
EpiF3-77
b
WT
EpiF3-68
EpiF3-77
EpiF3-15
c
Primary Root Length
Primary Root Length (mm)
P=0.003326
P=0.005531
n.s.
250
200
150
100
WT
EpiF3-68
EpiF3-15
EpiF3-77
d
Root Dry Weight
Root Dry Weight(g)
P=0.005531
P=0.017783
n.s.
0.03
0.02
0.01
WT
EpiF3-68
EpiF3-15
EpiF3-77
e
Plant Height
Plant Height (mm)
P=0.031872
n.s.
n.s.
175
150
125
100
WT
EpiF3-68
EpiF3-15
EpiF3-77
f
Aboveground Dry Weight
Aboveground Dry Weight(g)
P=0.032114
n.s.
n.s.
0.100
0.075
0.050
0.025
WT
EpiF3-68
EpiF3-15
EpiF3-77

Supporting figure 7

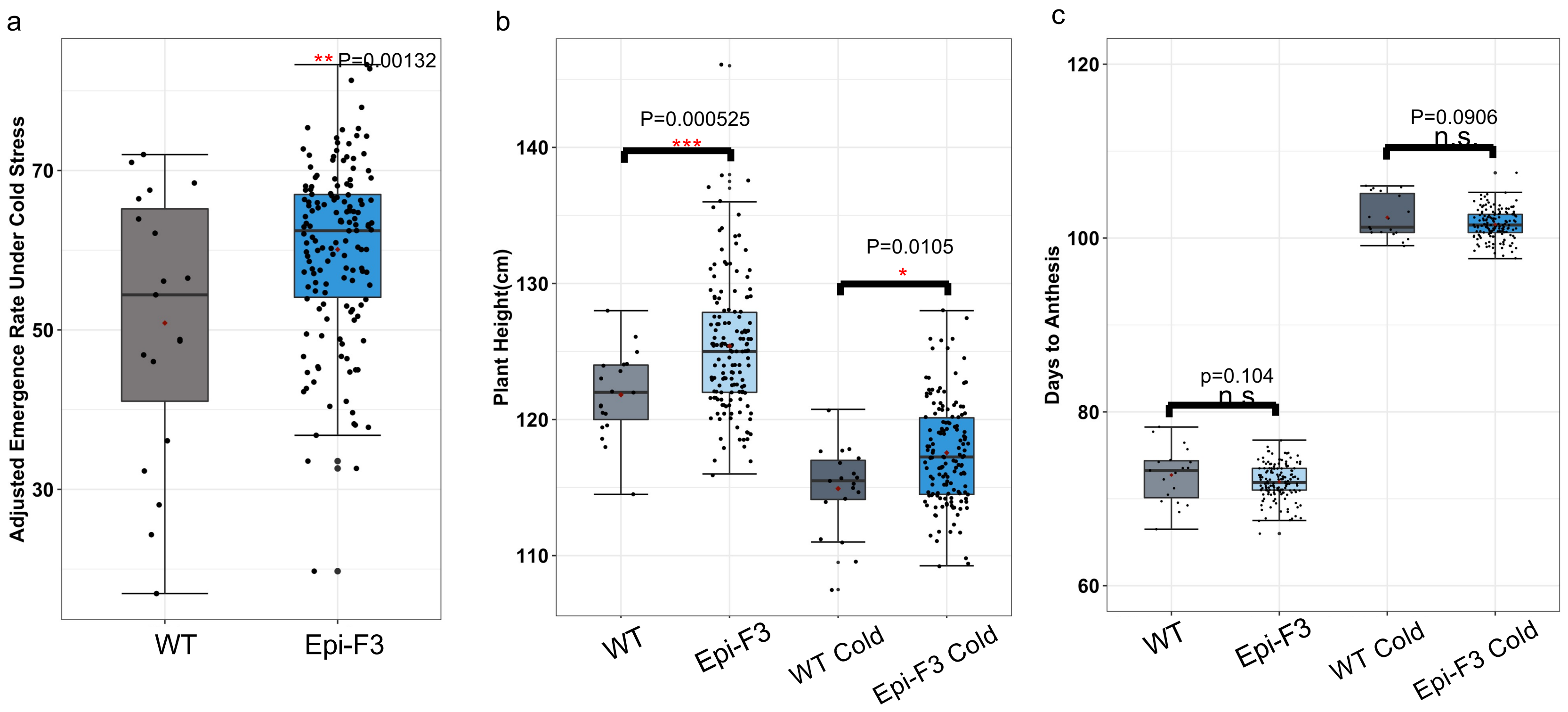

Supporting figure 8

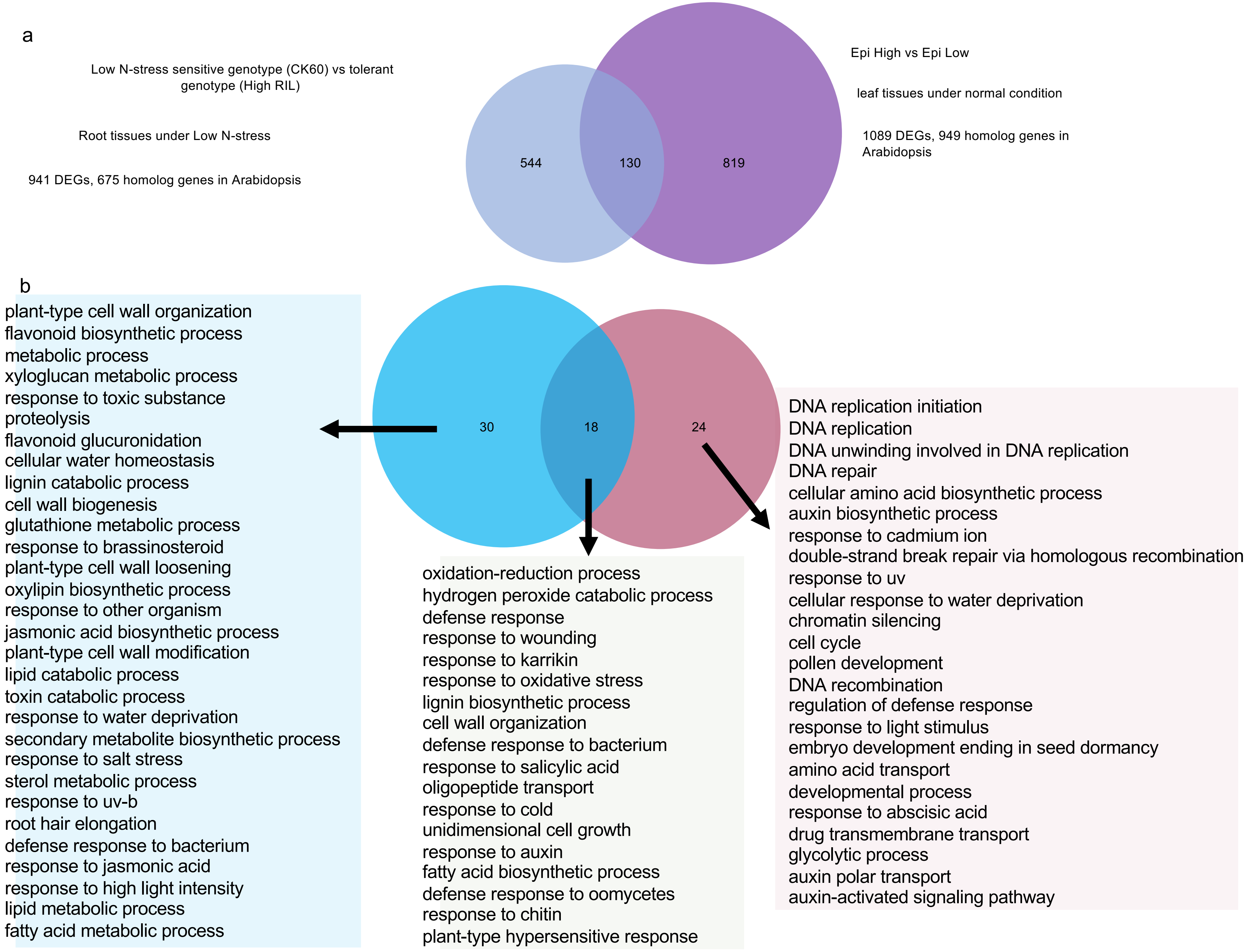

Supplement: Supplementary Figure 1 — Deriving epi-line materials in this study. (A) qRT-PCR analysis results for MSH1 transcript levels in variant phenotype plants with (+) and without (null) MSH1-RNAi transgene relative to wildtype Tx430. (B) Schematic diagram of the procedure for MSH1 suppression and de novo epi-line development for this study. [file Data_Sheet_1.pdf]
